# Investigation of aromatic compounds and olfactory profiles in cocoa pulp fermentation using yeast-based starters: A Volatilomics and machine learning approach

**DOI:** 10.1016/j.fochx.2025.102315

**Published:** 2025-02-25

**Authors:** Haode Chang, Chunhe Gu, Quanmiao Zhang, Wenjing Zhang, Liru Ma, Fei Liu, Zhen Feng

**Affiliations:** a Key Laboratory of Dairy Science, Ministry of Education, College of Food Science, Northeast Agricultural University, Harbin 150030, China; b Spice and Beverage Research Institute, Chinese Academy of Tropical Agricultural Sciences, Wanning 571533, Hainan, China; c Key Laboratory of Processing Suitability and Quality Control of the Special Tropical Crops, Wanning 571533, Hainan, China; d College of Life Sciences, Northeast Forestry University, Harbin 150006, China

**Keywords:** Cocoa, Fermentation, Machine learning, Aroma, GC–MS

## Abstract

The interaction and complex metabolism of microorganisms in cocoa pulp drive the fermentation process. To investigate this, four strains from spontaneous cocoa fermentation, including *Hanseniaspora uvarum, Saccharomyces cerevisiae, Lactiplantibacillus plantarum,* and *Gluconobacter potus* were combined to ferment cocoa pulp. Nineteen machine learning algorithms were run with the dataset of volatile compounds quantified by headspace solid-phase microextraction gas chromatography–mass spectrometry (HS-SPME-GC–MS) against integrated olfactory evaluation to reveal metabolite-sensory attribute relationships. The models showed high prediction accuracy, ranging from 0.85 for sourness by Gradient Boost Machine to 0.28 for sweetness by linear regression. Ethyl esters, specifically ethyl octanoate and ethyl 9-decenoate, were found positive for aroma development. Polynomial regression, neural network modeling and gradient boosting decision trees highlighted the high carbohydrate consumption rate of *S. cerevisiae*, the pectin degradation ability of *H. uvarum*, and the synergy of lactic acid bacteria with *G. potus*. This study offers new insights into cocoa flavor and the development of fermentation starter cocktails.

## Highlights

• 256 volatiles were identified and quantified by GC–MS in total.
• Random Forest and Gradient Boost Machine showed the best predictive performance.
• Ethyl esters contribute to desirable aroma in cocoa pulp fermentation.
• Lactic acid bacteria promote the rapid growth of *Gluconobacter potus* XY23.2.
• Fermentation with both *Saccharomyces* and *Hanseniaspora* exhibit better performance.

## 1 Introduction

Cocoa (*Theobroma cacao* L.) is a highly valuable tropical plant. Fresh cocoa beans usually undergo fermentation, drying, and roasting processes to obtain products such as cocoa powder and chocolate. Fermentation of cocoa beans is a complicated biochemical process involving a wide variability of microorganisms, including yeasts, lactic acid bacteria (LAB), and acetic acid bacteria (AAB) (De Vuyst & Leroy, 2020). The mucilaginous layer surrounding the cocoa beans, referred to as cocoa pulp, houses the fermentation process responsible for flavor and quality development in cocoa beans intended for chocolate production (Chetschik et al., 2018).

The fermentation process begins with yeasts fermenting the glucose in the pulp to ethanol and carrying out pectinolysis, aiding in pulp degradation. In this anaerobic stage, flavor compounds such as higher alcohols, aldehydes, organic acids, and esters are produced, contributing to the overall flavor profile of cocoa beans (Santander Munoz et al., 2020). The remaining sugars and citric acid are then fermented by LAB into lactic acid. This stabilizes the fermentation environment and provides lactate as a carbon source for AAB growth (Ho et al., 2015). AAB oxidize the ethanol produced by yeasts into acetic acid, which penetrates the bean cotyledons, inhibiting seed germination and initiating breakdowns of the seed subcellular structure (De Vuyst & Leroy, 2020).

The applications of cocoa pulp extend beyond its role in fermentation. It has been studied for aroma-active constituents that can influence the odor quality of fermenting cocoa beans. For example, specific varieties of cocoa pulp have been shown to be rich in odorants like 2-phenylethanol and 3-methylbutyl acetate, responsible for the floral and fruity notes in the final product (Bickel Haase et al., 2021; Chetschik et al., 2018). Cocoa pulp can be used as a natural sweetener and flavor enhancer in various food products, appreciated in its tropical and exotic flavor profiles. Cocoa pulp can also be used as a malt adjunct in beer production (Nunes et al., 2017) or as an ingredient in kefir beverages (Puerari et al., 2012). Cocoa honey and juice squeezed from mucilage effectively utilize cocoa's edible by-products (Guirlanda et al., 2021).

By employing gas chromatography combined with mass spectrometry and olfactometry techniques, it is possible to elucidate the metabolic profile and determine key metabolites. However, sensory evaluation is subjective and interactions between volatiles can influence the overall aroma perception, making the correlation between metabolites and sensory attributes challenging to evaluate (Niu et al., 2019). Various machine learning modeling approaches can assist in predicting the olfactometry characteristics of volatile organic compounds (VOCs) (Bi et al., 2020), and when integrated with metabolomics, they can reliably predict flavor attributes (Colantonio et al., 2022). These techniques can also help with peak picking and identification in mass spectrometry (Jirayupat et al., 2021).

The efficiency, accuracy, and reliability of food formulation and fermentation processes can be greatly improved by machine learning optimization techniques such as particle swarm optimization (PSO), genetic algorithms (GA), and neural networks (Liao et al., 2023; Mariano et al., 2010). Combining different machine learning regression and optimization methods can effectively control environmental factors to increase the yield of target substances during fermentation (Li et al., 2024). Furthermore, by controlling the fermentation process more precisely, these techniques can enhance the flavor quality of the final products (Chen et al., 2021).

This study aimed to investigate the impact of four microorganisms (*Hanseniaspora uvarum* XY23.1, *Saccharomyces cerevisiae* XY23.2, *Lactiplantibacillus plantarum* XY23.1 and *Gluconobacter potus* XY23.2) isolated and screened from the spontaneous fermentation of Hainan cocoa on cocoa pulp through 22 groups of mixture inoculation experiments. The study then utilized 19 distinct machine learning algorithms to assess the contribution of VOCs discovered from metabolomics to olfactometry attributes. Subsequently, combining characteristic metabolites and physiological indicators, predictive regression modeling and optimization algorithms were used to explore and enhance the cocoa fermentation process.

## 2 Materials and methods

### 2.1 Sample preparation

Cocoa pulp was sourced from Trinitario fine flavor cocoa in Hainan. The harvested cocoa pods were immediately cracked open with a machete, and the pulp was extracted and quickly transported to the laboratory. To prevent contamination and to maintain flavor characteristics (Bickel Haase et al., 2023), the pulp was preheated at 80 °C for 3 min and sterilized at 136 °C for 2 s using an integrated ultra-high temperature (UHT) sterilization unit (PT-20P, Trowin, China). The treated pulp was then rapidly cooled and homogenized. Finally, the cocoa pulp was stored in sterilized containers at −80 °C for future use.

### 2.2 Microbial strain preparation

On the basis of prominent flavor characteristics, four strains isolated from spontaneous cocoa fermentation were selected: *Hanseniaspora uvarum* XY23.1, *Saccharomyces cerevisiae* XY23.2, *Lactiplantibacillus plantarum* XY23.1, and *Gluconobacter potus* XY23.2. Yeast strains were revived in YPD medium at 28 °C for 24 h, *L. plantarum* was cultured in MRS medium at 37 °C for 24 h, and *G. potus* was cultured in GYC medium at 30 °C for 24 h, with over two generations. Each strain's viable cell count was measured by the relevant solid medium. The cultures were subsequently centrifuged at 5000 ×*g* for 15 min and washed with saline for further use.

### 2.3 Experimental design for the mixed fermentation

The mixed fermentation design of the four strains *Hanseniaspora uvarum* XY23.1 (X_1_), *Saccharomyces cerevisiae* XY23.2 (X_2_), *Lactiplantibacillus plantarum* XY23.1 (X_3_), and *Gluconobacter potus* XY23.2 (X_4_) was conducted via Design-Expert 13. The optimal design was employed with 15 model points, 4 lack-of-fit points, and 3 replicate points, totaling 22 runs. An essential confirmation of the presence of yeast in cocoa fermentation was constrained to at least 50 % yeast content in each experiment (Table 1).

**Table 1:** Mixture design for inoculated cocoa pulp fermentation and viable cell counts.

RUN No.	Fraction				Viable Cell Counts (LOG CFU/mL)						
	X_1_	X_2_	X_3_	X_4_		D0	D1	D2	D3	D4	D5
RUN01	61.31	15.63	0.00	23.06	Yeast	5.88	8.21 ± 0.21	8.29 ± 0.34	8.75 ± 0.64	7.14 ± 0.61	6.31 ± 0.29
					LAB	NA	NA	NA	NA	NA	NA
					AAB	5.36	4.47 ± 0.71	6.44 ± 0.48	8.09 ± 1.44	7.24 ± 0.55	6.38 ± 0.13
RUN02	61.03	0.00	31.11	7.86	Yeast	5.78	6.90 ± 1.30	5.07 ± 0.91	6.35 ± 0.21	4.64 ± 0.34	5.03 ± 0.38
					LAB	5.49	7.43 ± 0.85	7.99 ± 0.65	8.60 ± 0.59	6.00 ± 0.20	5.07 ± 0.91
					AAB	4.89	7.33 ± 0.46	7.92 ± 0.42	7.92 ± 0.42	6.60 ± 0.20	5.53 ± 1.37
RUN03	18.00	32.00	0.00	50.00	Yeast	5.7	8.58 ± 0.32	8.76 ± 0.58	8.44 ± 0.33	8.39 ± 0.73	7.10 ± 0.37
					LAB	NA	NA	NA	NA	NA	NA
					AAB	5.69	5.28 ± 0.75	5.69 ± 0.20	6.61 ± 0.28	7.48 ± 0.12	7.09 ± 0.36
RUN04	23.82	71.18	0.00	5.00	Yeast	5.98	7.96 ± 0.38	9.06 ± 0.52	8.41 ± 0.57	7.95 ± 0.42	5.63 ± 0.25
					LAB	NA	NA	NA	NA	NA	NA
					AAB	4.7	4.30 ± 0.10	6.04 ± 0.14	6.60 ± 0.21	7.30 ± 0.10	6.19 ± 0.33
RUN05	19.50	30.50	50.00	0.00	Yeast	5.7	7.76 ± 0.34	7.03 ± 0.53	7.93 ± 0.45	5.71 ± 0.27	4.41 ± 0.10
					LAB	5.7	7.67 ± 0.32	6.89 ± 0.21	6.81 ± 0.29	5.60 ± 0.21	4.59 ± 0.11
					AAB	NA	NA	NA	NA	NA	NA
RUN06	45.84	44.80	9.36	0.00	Yeast	5.96	7.75 ± 0.34	8.14 ± 0.31	8.36 ± 0.32	8.32 ± 0.28	7.99 ± 0.20
					LAB	4.97	6.34 ± 0.26	6.40 ± 0.30	6.10 ± 0.24	5.88 ± 0.15	4.76 ± 0.13
					AAB	NA	NA	NA	NA	NA	NA
RUN07	0.00	100.00	0.00	0.00	Yeast	6	7.76 ± 0.06	8.02 ± 0.12	8.30 ± 0.30	8.28 ± 0.27	8.25 ± 0.25
					LAB	NA	NA	NA	NA	NA	NA
					AAB	NA	NA	NA	NA	NA	NA
RUN08	61.31	15.63	0.00	23.06	Yeast	5.89	8.08 ± 0.18	8.20 ± 0.20	8.28 ± 0.37	7.60 ± 0.20	6.67 ± 0.20
					LAB	NA	NA	NA	NA	NA	NA
					AAB	5.36	4.85 ± 0.35	6.70 ± 0.30	6.61 ± 0.13	7.43 ± 0.41	6.54 ± 0.34
RUN09	100.00	0.00	0.00	0.00	Yeast	6	8.32 ± 0.32	8.00 ± 0.15	6.79 ± 0.59	6.48 ± 0.28	6.01 ± 0.11
					LAB	NA	NA	NA	NA	NA	NA
					AAB	NA	NA	NA	NA	NA	NA
RUN10	50.00	0.00	0.00	50.00	Yeast	5.7	6.37 ± 0.92	6.16 ± 0.43	5.48 ± 0.71	4.00 ± 0.16	NA
					LAB	NA	NA	NA	NA	NA	NA
					AAB	5.7	6.04 ± 0.13	6.72 ± 0.43	3.95 ± 0.42	3.93 ± 0.14	3.94 ± 0.11
RUN11	33.88	16.12	21.08	28.92	Yeast	5.7	8.38 ± 0.41	7.88 ± 0.50	8.50 ± 0.10	7.32 ± 0.15	6.87 ± 0.87
					LAB	5.32	7.38 ± 0.21	6.86 ± 0.33	6.30 ± 0.67	6.19 ± 0.03	5.82 ± 0.95
					AAB	5.46	7.38 ± 0.20	6.58 ± 0.98	6.32 ± 0.08	6.68 ± 0.77	6.55 ± 0.02
RUN12	0.00	62.04	0.00	37.96	Yeast	5.79	8.10 ± 0.69	8.61 ± 0.36	8.34 ± 0.42	8.00 ± 0.30	6.19 ± 0.03
					LAB	NA	NA	NA	NA	NA	NA
					AAB	5.58	4.48 ± 0.71	5.82 ± 0.95	6.31 ± 0.87	6.15 ± 0.23	5.81 ± 0.29
RUN13	0.00	73.87	26.13	0.00	Yeast	5.87	7.91 ± 0.85	8.00 ± 0.43	8.20 ± 0.19	8.20 ± 0.33	7.74 ± 0.54
					LAB	5.42	7.25 ± 0.25	6.72 ± 0.20	6.42 ± 0.49	6.32 ± 0.81	6.04 ± 0.37
					AAB	NA	NA	NA	NA	NA	NA
RUN14	45.84	44.80	9.36	0.00	Yeast	5.96	7.83 ± 0.25	7.25 ± 0.04	8.46 ± 0.09	8.42 ± 0.32	8.30 ± 0.67
					LAB	4.97	6.69 ± 0.20	6.23 ± 0.30	6.05 ± 0.49	5.65 ± 0.32	5.67 ± 0.21
					AAB	NA	NA	NA	NA	NA	NA
RUN15	9.95	40.05	25.78	24.22	Yeast	5.7	8.25 ± 0.29	6.79 ± 0.03	8.12 ± 0.18	7.31 ± 0.54	5.54 ± 0.16
					LAB	5.41	7.38 ± 0.20	6.32 ± 0.43	6.49 ± 0.14	5.45 ± 0.72	5.00 ± 0.14
					AAB	5.38	7.45 ± 0.42	6.65 ± 0.32	6.28 ± 0.25	6.71 ± 0.93	6.27 ± 0.72
RUN16	61.03	0.00	31.11	7.86	Yeast	5.79	7.31 ± 0.54	6.38 ± 0.70	6.70 ± 0.89	5.77 ± 0.45	4.58 ± 0.28
					LAB	5.5	6.60 ± 0.21	7.26 ± 0.58	7.45 ± 0.72	6.95 ± 0.42	5.66 ± 0.38
					AAB	4.9	6.70 ± 0.89	7.22 ± 0.87	7.48 ± 0.71	7.11 ± 1.39	6.07 ± 0.82
RUN17	0.00	52.50	47.50	0.00	Yeast	5.71	8.28 ± 0.46	8.01 ± 0.56	7.93 ± 0.34	7.89 ± 0.41	7.73 ± 0.29
					LAB	5.65	7.65 ± 0.35	7.32 ± 0.35	6.99 ± 0.12	6.81 ± 0.61	6.74 ± 0.44
					AAB	NA	NA	NA	NA	NA	NA
RUN18	41.59	8.41	24.40	25.60	Yeast	5.7	8.00 ± 0.41	7.84 ± 0.39	7.83 ± 0.26	7.86 ± 0.57	7.37 ± 0.11
					LAB	5.34	7.37 ± 0.11	6.86 ± 0.33	6.85 ± 0.51	5.31 ± 0.54	3.89 ± 0.21
					AAB	5.4	7.39 ± 0.87	7.41 ± 0.08	6.97 ± 0.73	7.03 ± 0.25	6.84 ± 0.38
RUN19	47.28	25.82	26.90	0.00	Yeast	5.79	8.19 ± 0.93	8.06 ± 0.60	8.14 ± 0.36	7.92 ± 0.42	5.93 ± 0.29
					LAB	5.49	7.19 ± 0.83	7.20 ± 0.95	6.49 ± 0.15	5.81 ± 0.29	4.95 ± 0.09
					AAB	NA	NA	NA	NA	NA	NA
RUN20	34.66	41.64	0.00	23.70	Yeast	5.89	8.27 ± 0.18	8.29 ± 0.53	8.37 ± 0.55	7.31 ± 0.50	7.27 ± 0.17
					LAB	NA	NA	NA	NA	NA	NA
					AAB	5.32	6.40 ± 0.97	6.54 ± 0.38	7.27 ± 0.49	7.03 ± 0.34	6.65 ± 0.32
RUN21	6.05	67.95	12.89	13.11	Yeast	5.72	8.35 ± 0.42	7.76 ± 0.29	8.41 ± 0.13	8.39 ± 0.47	8.24 ± 0.30
					LAB	5.19	7.10 ± 0.37	6.82 ± 0.95	6.44 ± 0.38	6.10 ± 0.04	5.02 ± 0.21
					AAB	5.13	7.25 ± 0.04	7.32 ± 0.20	7.33 ± 0.24	7.34 ± 0.59	6.54 ± 0.28
RUN22	23.09	49.23	27.68	0.00	Yeast	5.98	8.24 ± 0.05	8.31 ± 0.18	9.34 ± 0.40	8.19 ± 0.93	7.99 ± 0.86
					LAB	4.92	7.33 ± 0.12	7.32 ± 0.20	7.25 ± 0.24	6.81 ± 0.62	6.74 ± 0.44
					AAB	NA	NA	NA	NA	NA	NA

For each of the 22 experimental designs, the microbial strains prepared as described in Section 2.2 were mixed at the specified ratio and inoculated into 250 mL of sterile cocoa pulp to ensure a microbial concentration of 6 log CFU/mL. Fermentation was carried out in an incubator at 30 °C for 5 days, with daily sampling. The experiment was conducted in triplicate for each run.

### 2.4 Microbial and chemical analyses

#### 2.4.1 Viable cell count

Yeasts were counted on YPD agar supplemented with 0.01 % chloramphenicol and incubated at 28 °C for 48 h. LAB were counted on MRS agar supplemented with 0.01 % natamycin and incubated at 37 °C for 48 h. AAB were cultured on acetic acid bacteria agar (containing 1 % glucose, 1 % yeast extract, 2 % agar and 30 mL/L ethanol) supplemented with 0.01 % natamycin and incubated at 30 °C for 72 h.

#### 2.4.2 Total polyphenols

The total polyphenol content (TPC) was determined by Folin-Ciocalteu method using gallic acid as a standard (Miller et al., 2008). Cocoa pulp (1 g) was extracted with a mixture of acetone, water, and acetic acid in a 70.0:29.5:0.5 volume ratio by sonication for 10 min, followed by centrifugation at 6000 ×*g* for 5 min. The supernatant (1 mL) was reacted with Folin-Ciocalteu reagent (100 μL) and sodium carbonate solution (20 %, *w*/*v*, 3 mL). After 30 min of incubation, the absorbance was measured at 765 nm using a microplate spectrophotometer (Bio-Rad, iMark680, Hercules, California, USA).

#### 2.4.3 Pectinolytic activity

Pectinolytic activity was determined by measuring the amount of reducing groups released due to the degradation of polygalacturonic compounds, using the dinitrosalicylic acid (DNS) method (da Silva et al., 2005). Galacturonic acid (GA) was made as a standard.

#### 2.4.4 HPLC

Citric acid, lactic acid, acetic acid, glucose, fructose, and ethanol contents were measured using a high-performance liquid chromatography (HPLC) instrument (Waters, USA) with an Xtimate Sugar-H column (Welch Materials, China) and a differential refractometer detector.

### 2.5 VOCs analysis by HS-SPME-GC–MS

Each sample of cocoa pulp (5.0 mL) was added to headspace vials (20 mL) for headspace solid-phase microextraction (HS-SPME). 10 μL of 2-octanol (20 mg/L) was added to each sample as an internal standard. Samples were analyzed using an Agilent 7890B gas chromatograph coupled with an Agilent 5977B mass selective detector (Agilent Technologies, Santa Clara, CA, USA). The extraction of flavor compounds was performed using a DVB/CAR/PDMS fiber inserted into a preheated sample bottle for 30 min. Subsequently, the fiber was desorbed in the GC–MS system. A DB-WAX capillary column (30 m × 250 μm × 0.25 μm, Agilent Technologies, USA) was used in the GC, with the following temperature program: starting at 40 °C for 5 min, ramping to 180 °C at 4 °C/min, and finally ramping to 260 °C with a 2-min hold. Helium served as the carrier gas at a constant flow rate of 1 mL/min.

The MS detector operated in electron impact mode at 70 eV ionization energy and scanned the mass range of 20–500 *m*/*z*. The ion source temperature was maintained at 260 °C, while the quadrupole mass detector transfer line temperature was kept at 180 °C. Identification of flavor compounds was based on comparison of their mass spectra with those in the NIST2017 library (Agilent).

### 2.6 Sensory evaluation

The olfactory evaluations were conducted following the methods of Zarzo (2015) and Bickel Haase et al. (2023). Ethical permission for sensory research was not required in our institution (Northeast Agricultural University and Spice and Beverage Research Institute). Trained assessors were chosen from a group of 22 people trained for two periods of 15 days each within a period of six months. The training involved assessing sensitivity, descriptive ability, and repeatability using various concentrations of compounds and juices absorbed in odorless cotton wool. Based on their performance, 11 panelists (4 males and 7 females) were selected for the sensory evaluation of fermented cocoa pulp.

To investigate the impact of microorganisms and ensure discriminative grouping of sensory evaluation data, the following attributes were assessed: sweetness (including sweet, caramel, chocolate, honey, slightly fruity characteristics), sourness (including pungent, acidy, fruity, lactic, citrusy characteristics), herbaceous (including fresh, green, citrusy, plant-like characteristics), savory (including cheesy, fatty, umami-like characteristics), and overall impression representing liking (Supplementary material S1). A total of 110 samples of fermented pulp were evaluated.

Samples were divided into three portions and, at different times, evaluated by different assessors in order to ensure data accuracy. Each assessor rated up to 20 samples per day, by assigning a score from 0 to 100 for each attribute. All data were standardized using z-scores for further analyses.

To be noted, all participants in the sensory evaluation study provided informed consent prior to participation, in accordance with ethical guidelines and regulations governing human sensory research.

### 2.7 Modeling and optimization methods

Spearman correlation analysis was used to explore the relationships between metabolites and olfactory characteristics due to its robustness to non-normal distributions and outliers commonly present in omics and sensory data. Subsequently, 19 regression modeling methods were applied: Linear Regression, Bayesian Ridge Regression (BRR), Least Absolute Shrinkage and Selection Operator (LASSO), Bayesian LASSO, Elastic Net, Support Vector Machine (SVM) Linear, SVM Radial, SVM Radial Sigma, Relevance Vector Machine (RVM), Reproducing Kernel Hilbert Space (RKHS), Kernel Partial Least Squares Regression (KPLS), Multilayer Perceptron Neural Network (MLP), Bayesian Neural Network (BNN), Random Forest (RF), Gradient Boosting Machines (GBM), eXtreme Gradient Boosting (XGBoost), Bayes A, Bayes B and Bayes C. These methods modeled the relationship between VOC content data and olfactory ratings.

To evaluate the prediction performance of each model, a 10-fold holdout cross-validation was employed (Colantonio et al., 2022). The dataset, consisting of 110 samples, was randomly divided into 10 equal subsets. In each of the 10 iterations, 9 subsets were used to train the model, while the remaining subset served as the test set. A secondary 10-fold cross-validation within the training set was performed to fine-tune hyperparameters by minimizing root-mean-squared error (RMSE). The optimized models were then applied to the primary test set to predict flavor ratings, and the correlation between predicted and observed ratings was calculated for each iteration. The average correlation across all 10 iterations represented the model's overall accuracy.

By identifying models with high accuracy, GBM and RF were utilized to find key metabolites. These key metabolites and chemical indices (responses) were modeled against 22 fermentation starter ratios (inputs). We employed multiple approaches for modeling to better charactize the relationships: (1) polynomial models, as it is a standard approach for mixture experiments and higher-order terms allow these models to approximate interactions among components; (2) multilayer perceptron neural network (MLP) for its ability to model complex, non-linear relationships without requiring a predefined functional form and (3) Gradient Boosting Decision Trees (GBDT) as a data-driven approach that captures complex patterns without needing assumptions about the relationships between variables. These modelings were performed in JMP Pro 17. Model quality was assessed comprehensively using analysis of variance (ANOVA) or the coefficient of determination (R^2^). Given the complex relationships between strain ratios and multiple response variables in fermentation processes, GA was employed to optimize the polynomial and MLP models with good predictive ability (Scrucca, 2013). GBDT was then used to predict the responses under the optimized conditions obtained from GA, providing an additional reference for evaluating the optimization results.

### 2.8 Statistical analyses

Apart from the methods mentioned above, the application and accuracy of machine learning algorithms for VOCs and olfactory attributes, as well as the calculation of MSE and weights, were all performed using the corresponding packages in R 4.4.0. GA was executed using the GA package in R 4.4.0. All plots were created using ggplot2 and additional plugin packages. The database involved in model establishment can be found in Supplementary Material S2.

## 3 Results

### 3.1 Microbial colony dynamics and metabolic patterns

**Fig. 1:**
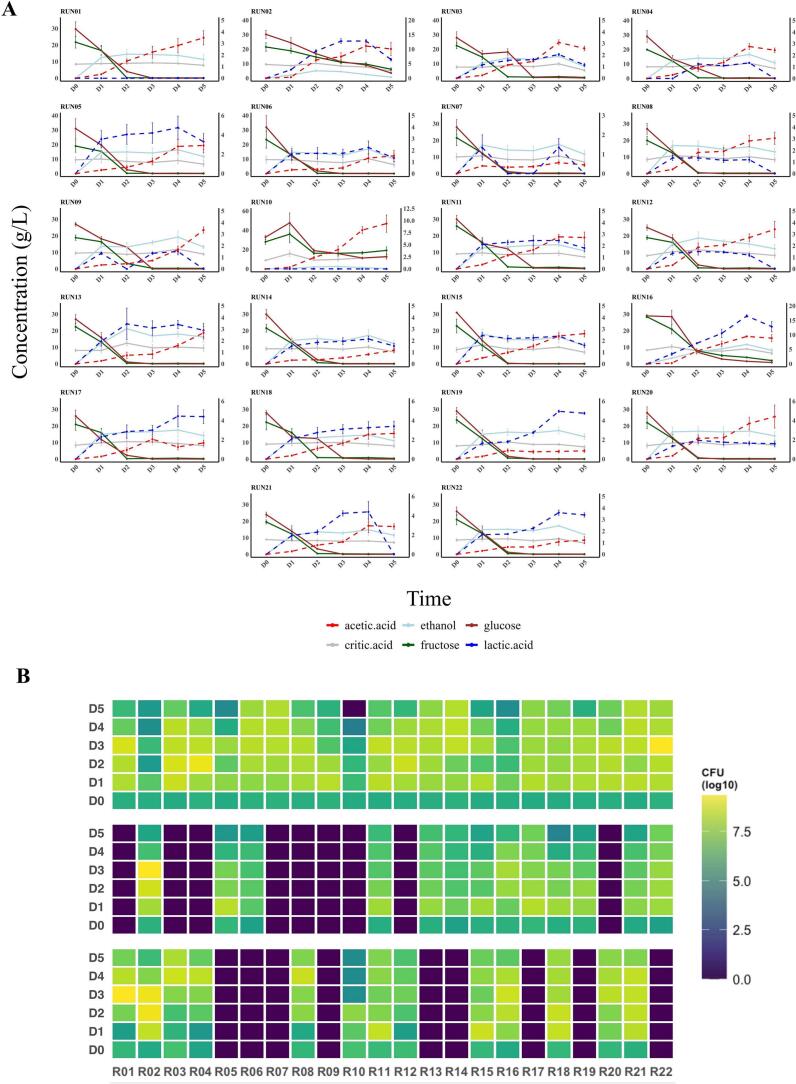
(A) Changes in metabolite concentrations across 22 runs analyzed by HPLC. The left Y-axis and solid lines represent ethanol, glucose, citric acid, and fructose, while the right Y-axis and dashed lines represent acetic acid and lactic acid. (B) Heatmap of viable cell counts from top to bottom representing yeasts, LAB, and AAB during fermentation (D0-D5).

*H. uvarum* XY23.1 (X1), *S. cerevisiae* XY23.2 (X2), *L. plantarum* XY23.1 (X3), and *G. potus* XY23.2 (X4) were inoculated into sterile cocoa pulp at a total concentration of 6 LOG CFUs, divided into 22 groups, for a 5-day fermentation period (D0-D5). Samples were taken daily, serially diluted, and plated on selective solid media to observe microbial growth trends (Table 1). Except for the 2nd, 10th, and 16th runs, all the other groups presented high colony counts on the first day. Interestingly, *G. potus* grew more slowly in the groups where L. *plantarum* was not involved. HPLC was used to observe the microorganisms' sugar consumption capacity and basic metabolic performance (Fig. 1). Lower ethanol yields and slower sugar metabolism were also observed in the 2nd, 10th, and 16th runs. There was little change in the amount of citric acid during the fermentation process. By the third day, most groups had run out of glucose and fructose, accompanied by a gradual increase in acetic acid and ethanol contents. By the fifth day, a progressive drop in yeast metabolic activity was evident from the rise in acid and decrease in ethanol.

### 3.2 A first glimpse into VOCs and spearman correlation analysis

**Fig. 2:**
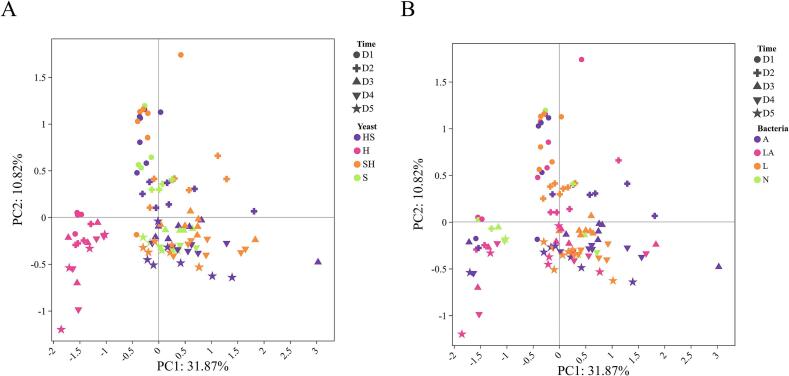
Principal component analysis of VOC profiles during inoculated cocoa pulp fermentation across 22 experiments. (A) Categorized by yeast: H indicates inoculation with *Hanseniaspora* only, S with *Saccharomyces* only, HS indicates both present with a higher proportion of *Hanseniaspora*, SH indicates both present with a higher proportion of *Saccharomyces*; (B) Categorized by bacteria: A indicates inoculation with *Gluconobacter* only, L with *Lactiplantibacillus* only, LA indicates both present, and N indicates no bacterial inoculation.

**Fig. 3:**
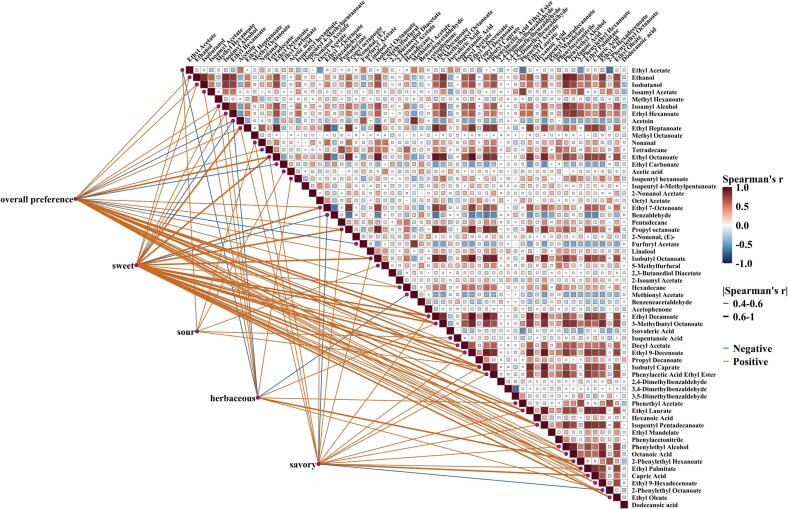
Spearman correlation analysis of VOCs and olfactory attributes in cocoa pulp fermentation. Only correlations with |r| > 0.4 and p < 0.05 are shown for the association between VOCs and olfactory attributes.

To evaluate VOCs' contribution to olfactory attributes through machine learning algorithms, we first established a comprehensive VOC profile (Supplementary material S2). The samples collected every 24 h revealed strong and distinct aromas. Aroma description and VOC analysis was conducted on a total of 110 samples involved in the fermentation. A HS-SPME-GC–MS was used to identify and quantify a total of 259 volatiles. After eliminating metabolites that did not exhibit variability in this population, 68 VOCs were left from the original set to avoid excessive zero values influencing the subsequent modeling. These included 8 carboxylic acids, 6 alcohols, 10 aldehydes, 3 ketones, and 36 esters. Principal component analysis (PCA) was applied to transform the multidimensional data into a smaller set of uncorrelated principal components, revealing that the VOC distribution was significantly different in the group inoculated only with Hanseniaspora compared to other groups (Fig. 2). A Spearman correlation analysis was performed to jointly analyze these VOCs and sensory attributes, showing significant moderate (0.4–0.6) and strong (0.6–1.0) correlations (*p* < 0.05) between aroma descriptions and VOCs (Fig. 3, Fig. S1). Larger esters and medium-chain fatty acids in particular showed moderate (*r* = 0.4–0.6, p < 0.05) to strong (*r* > 0.6, *p* < 0.01) positive correlations with sweetness and overall preference, whereas ethyl carbonate, furfuryl acetate, acetoin, and benzaldehyde might be off-flavoring agents (*r* < −0.4, p < 0.05). These compounds did, however, also exhibit inverse relationships with the majority of other esters.

### 3.3 Predicting olfactory attributes with volatile metabolites

**Fig. 4:**
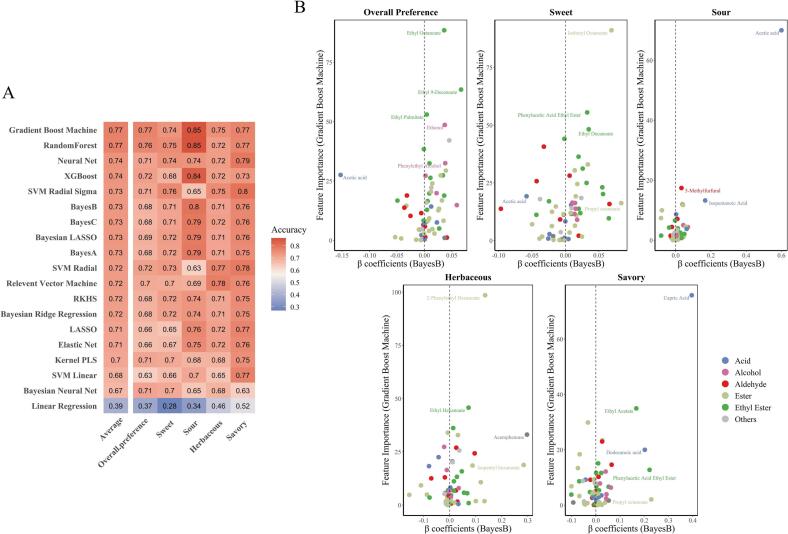
(A) Comparison of machine learning models for predicting sensory attributes in cocoa pulp fermentation; (B) Importance of metabolites on olfactory attributes based on feature importance from Gradient Boost Machine (*y*-axis) and β coefficients from BayesB (*x*-axis).

To capture complex, multi-dimensional relationships between metabolites and sensory characteristics, 19 machine learning methods were employed, using the content of VOCs as input and aromatic traits as the response for modeling. Each model underwent 10-fold validation, with the average value representing the final accuracy (Fig. 4A, Table S1). Due to the ambiguous and highly subjective nature of the overall preference evaluation metric, the overall accuracy of different models was relatively low (0.68). The models predicted sour and savory attributes more accurately (0.73 and 0.74) due to their distinct olfactory characteristics. Ensemble methods (GBM, Random Forest, XGBoost) demonstrated outstanding predictive capabilities, especially for the sour attribute. Neural networks also exhibited high average accuracy (0.74). Specifically, LASSO and Elastic Net showed poor explanatory power for the sweet attribute (0.65 and 0.67). Linear Regression (0.39) lacked explanatory power for all attributes. For complex olfactory traits, models such as GBM, Random Forest, Neural Networks, and XGBoost have demonstrated reliable predictions based on metabolite data.

### 3.4 Digging up key VOCs during fermentation

To identify the VOCs that contribute most significantly to olfactory perception, we attempted to use models with high predictive accuracy from the 10-fold cross-validation results. GBM demonstrated the highest accuracy (0.85 in average) for sour attributes, while RF showed similar performance (0.83 in average), making them primary candidates for feature importance analysis. However, both RF and GBM are based on decision trees and do not directly provide the direction of the relationship in this study. To better illustrate the relative importance of each metabolite, we used the feature importance from GBM and the β coefficients from the relatively best-performing Bayesian model, BayesB, for subsequent analysis (Fig. 4B). This combination allowed us to rank VOCs by their importance using GBM and also understanding their directional relationships with olfactory attributes through BayesB's β coefficients.

Through comprehensive evaluation of the two models, the strong acidic characteristic of acetic acid was found to have a negative impact on overall aroma development, while ethyl esters such as ethyl octanoate, ethyl 9-decenoate, and ethyl palmitate, along with alcohols, had a more positive influence on overall preference. For sweet characteristics, ethyl esters (phenylacetic acid ethyl ester and ethyl decanoate) produced from the reaction of amino acids and ethanol during yeast fermentation were the main contributors. The esterification of isobutanol and octanoic acid was also considered to significantly contribute to the sweet aroma. Acetic acid naturally had a significant impact on acidic flavor characteristics, but 5-methyl furfural, usually associated with caramel flavor, was also found to have a high positive contribution. For the more complex herbaceous flavor, GBM inferred that 2-phenylethyl hexanoate greatly contributed to the green, fresh aroma, with isopentyl hexanoate and acetophenone having β coefficients close to 0.3. Medium-chain fatty acids (MCFAs), especially carpic acid, and ethyl esters (phenylacetic acid ethyl ester and ethyl acetate) that also contribute to sweet aroma appeared to positively enhance savory attributes. It is evident that VOCs have varying impacts on olfactory perception, and their development and interactions are crucial for flavor construction.

### 3.5 Modeling attempts using polynomial and neural networks

Following the identification of key VOCs and their sensory impacts, multiple modeling approaches were employed to determine optimal strain combinations for desired fermentation outcomes. As response variables, we selected several key parameters: the overall preference score at fermentation end, acetic acid content affecting sour characteristics, esters important for flavor development (including their subset ethyl esters), ethyl 9-hexadecenoate and ethyl octanoate (OPEE) for their strong impact on preference, pectinase activity, and total polyphenol content. Except for overall preference, the average values during fermentation were chosen for prediction, taking into account the dynamic changes in the metabolic process.

Polynomial models were evaluated sequentially based on the R^2^, *p*-value, and Lack of Fit F-value obtained from ANOVA, following the order of linear, quadratic, special cubic, and cubic. Polynomial models for ethanol and OPEE were not considered (R^2^ < 0.4). The special cubic model could better predict acetic acid content (R^2^ = 0.92) and pectinase activity (R^2^ = 0.93) (*p* < 0.05) (Table 2, Eqs. (1–2)), although the special cubic model had good explanatory power for polyphenols (R^2^ = 0.91). The Lack of Fit F-value of 10.35 implies the Lack of Fit is significant.

(1) $$Y\;\left(\text{acetic acid content},g/L\right)=0.00883287^{*}X_{1}+0.0758676^{*}X_{2}+0.100776^{*}X_{3}+0.931215^{*}X_{4}+0.491804^{*}{X_{1}}^{*}X_{2}+3.53252^{*}{X_{1}}^{*}X_{3}+0.481629^{*}{X_{1}}^{*}X_{4}–0.258^{*}{X_{2}}^{*}X_{3}–1.58824^{*}{X_{2}}^{*}X_{4}–15.4147^{*}{X_{3}}^{*}X_{4}–12.6093^{*}{X_{1}}^{*}{X_{2}}^{*}X_{3}–6.99338^{*}{X_{1}}^{*}{X_{2}}^{*}X_{4}+13.063^{*}{X_{1}}^{*}{X_{3}}^{*}X_{4}+37.173^{*}{X_{2}}^{*}{X_{3}}^{*}X_{4}$$

(2) $$Y\;\left(\text{pectinase},g\;\left(\text{galacturonic acid}\;\mathrm{eqv}.\right)/L\right)=4.98185^{*}X_{1}+2.81485^{*}X_{2}+8.00902^{*}X_{3}+48.106^{*}X_{4}+5.54099^{*}{X_{1}}^{*}X_{2}+39.308^{*}{X_{1}}^{*}X_{3}–8.81131^{*}{X_{1}}^{*}X_{4}–18.8789^{*}{X_{2}}^{*}X_{3}–76.1764^{*}{X_{2}}^{*}X_{4}+86.3545^{*}{X_{3}}^{*}X_{4}–202.544^{*}{X_{1}}^{*}{X_{2}}^{*}X_{3}–328.029^{*}{X_{1}}^{*}{X_{2}}^{*}X_{4}–538.889^{*}{X_{1}}^{*}{X_{3}}^{*}X_{4}–65.7449^{*}{X_{2}}^{*}{X_{3}}^{*}X_{4}$$

**Table 2:** The coefficient of determination (R^2^), RMSE, and predicted values of the three models for the selected responses.

Response	Models									
	Polynomial				Neural Network			GBDT		
	R^2^	Type	p-Value	Predicted Value	R^2^	RMSE	Predicted Value	R^2^	RMSE	Predicted Value*
Overall Preference	**0.86**	**Special Cubic**	**0.05**	**76.86**	**0.76**	**6.58**	**67.13**	0.8	5.81	67.61 (for polynomial model) and 59.11 (for MLP)
Acetic Acid	**0.92**	**Special Cubic**	**0.01**	**0**	**0.96**	**0.04**	**0**	0.96	0.04	0.03 (for polynomial model) and 0.01 (for MLP)
Ester	0.68	Special Cubic	0.45	Not significant	0.76	0.94	NA	NA	NA	NA
Ethyl Ester	0.77	Special Cubic	0.21	Not significant	**0.83**	**0.67**	**8.78**	0.85	0.64	6.59
OPEE	0.31	Linear	0.09	Not significant	**0.89**	**0.34**	**2.72**	0.9	0.33	2.37
Ethanol	0.36	Linear	0.05	Not significant	0.72	0.52	NA	NA	NA	NA
Pectinase Activity	**0.93**	**Special Cubic**	**0.01**	**18.72**	**0.84**	**1.76**	**s14.25**	0.81	2.12	10.58 (for polynomial model) and 11.26 (for MLP)
Total Polyphenol	0.91	Special Cubic	0.02	Lack of Fit	**0.91**	**1.3**	**8.92**	0.88	1.93	7.26
	*The predicted value of GBDT is used to validate the optimal predictions of polynomial and/or MLP. The bold blocks indicate that the corresponding models were selected for GA optimization									

A perceptron neural network with one hidden layer and four neuron nodes activated by the hyperbolic tangent function (NTanH) was used for the same inputs and responses (Table 2). After 5-fold cross-validation, it was found that the MLP could better predict acetic acid (R^2^ = 0.96, RMSE = 0.04). In addition, the model quality for esters (R^2^ = 0.76, RMSE = 0.94) and ethyl esters (R^2^ = 0.83, RMSE = 0.67) was improved, and the prediction ability for EEOP (R^2^ = 0.89, RMSE = 0.34) also significantly improved when compared to the polynomial model.

### 3.6 GA optimization and GBDT validation for cocoa pulp fermentation

Multiple high-quality models were used for GA optimization to determine the ideal optimal solution following the above regression analysis. Except for acetic acid, which seeks a minimum value, all other indicators aim for maximization (Table 2). GA was set with a population size of 50, a maximum of 500 iterations, and a stop criterion of no improvement for 50 consecutive generations. GA offered a solution with a relatively even distribution (x(1):x(2):x(3):x(4) = 32.44:34.71:15.14:17.71) of the four strains for the cubic model that targeted overall preference, whereas the MLP model highlighted the role of yeasts (x(1):x(2):x(3):x(4) = 44.55:31.52:24.88:13.06). Although distinct solutions were offered for the neural network model and the special cubic model aimed at AAB, the best response in each scenario was both zero. To increase the yield of ethyl ester, ethyl 9-hexodecenoate, and ethyl octanoate, *H. uvarum* XY23.1 was speculated to be more necessary among the four strains.

To strengthen the validation of our optimization results, we employed a comprehensive cross-model validation approach. The GBDT)model, which demonstrated robust performance in metabolite prediction (R^2^ ranging from 0.80 to 0.96), was used to validate the optimal solutions obtained from both polynomial regression and MLP models. For key response variables like overall preference, acetic acid content, and pectinase activity, the GBDT predictions showed strong alignment with GA-optimized values (Table 2). Notably, for ethyl esters and OPEE (ethyl octanoate and ethyl 9-decenoate), where GBDT achieved high model quality (R^2^ = 0.85 and 0.90 respectively), the predicted yields were consistently within 25 % of the GA-optimized values from MLP modeling. These optimization results, supported by GBDT validation, provided specific guidance for enhancing cocoa fermentation through optimal strain ratios, thereby achieving our goal of process optimization. For reference, if the goal is to meet the response targets of the cubic models, DE provided the result of x(1):x(2):x(3):x(4) = 30.48:43.04:5.95:20.53.

## 4 Discussion

In this study, we employed machine learning approaches to investigate aroma development in cocoa pulp during fermentation, successfully establishing relationships between VOCs and olfactory attributes. Through a series of regression and modeling methods, the contribution of microorganisms (yeasts, LAB and AAB) in the fermentation process was evaluated, developing ideal formula for cocoa starter cocktails. Cocoa pulp provides sugars and nutrients essential for the fermentation process, which is characterized by a succession of yeasts, LAB, and AAB. The edibility of cocoa pulp itself, along with its rich content of low-degradation-rate fiber oligosaccharides and polyphenol oxidase, means it is a valuable source of bioactive substances (Moretti et al., 2023). By elucidating the direct impact of microorganisms on cocoa mucilage, we can not only better understand the contributions of these microbes but also clarify key flavor properties in the pulp. Leveraging these insights, we may be able to apply and optimize a new fermentation method for cocoa beans that is comparable to the honey (semi-dry) processing for coffee (de Melo Pereira et al., 2019). This approach might make full use of the to-be-discarded pulp, helping to reduce waste and pollution.

Starters are typically discovered by screening local strains isolated from cocoa-producing countries or by using commercial strains isolated from other sources (Assi-Clair et al., 2019; Diaz-Munoz & De Vuyst, 2022). Dominant and high-performing strains isolated from natural fermentation are often used as candidates, and some studies have adopted breeding strategies to obtain desirable characteristics (Meersman et al., 2017; Miguel et al., 2017). Our multi-strain optimization approach aligns with the emerging trend toward designed starter cultures in a new statistical way and analyze microbe's contribution deeply.

Yeasts play a crucial role in the quality of cocoa fermentation via their pectinolytic and metabolic properties (Ho et al., 2014). Experiments using *H. uvarum* XY23.1 and *S. cerevisiae* XY23.2 revealed high levels of alcohols (isoamyl alcohol, isobutanol and phenylethyl alcohol). These alcohols react with medium-chain fatty acids and acetic acid to produce rich ethyl and acetate esters, thereby influencing the final aroma quality of cocoa products (De Vuyst & Leroy, 2020; Diaz-Munoz et al., 2023). However, different growth trends were observed in cocoa pulp with varying ratios of the two yeast strains. Runs containing only *H. uvarum* demonstrated higher pectinase activity expression and slower growth rates due to *Hanseniaspora*'s low sugar consumption rate (Valera et al., 2021). In contrast, mixed cultures of *H. uvarum* and *S. cerevisiae* demonstrated rapid yeast proliferation. While pectinase activity was lower in these mixed runs, the liquefaction rate of the pulp seemed to remain consistent across all groups. It is likely that *H. uvarum* interacted with *S. cerevisiae* not only through cell-to-cell contact (Renault et al., 2013) but also via metabolic activation targeting glucose and nitrogen metabolism, enhancing competitiveness of *S. cerevisiae* (Curiel et al., 2017). Additionally, *H. uvarum* can elevate the concentration of VOCs through β-glucosidase activity, while producing relatively lower levels of organic acids compared to other non-*Saccharomyce*s yeasts (Hu et al., 2018). The presence of multi yeasts during fermentation can lead to a distinctive volatile profile that contrasts with single-yeast fermentations. Batista et al. (2016) found that synergy effects among *S. cerevisiae*, *P. kluyveri*, and *H. uvarum* reduced the astringency of dried cocoa beans while enhancing bitterness and cocoa flavor intensity.

An acidic environment is established by aerobic AAB and anaerobic LAB, conducive to the production of abundant flavor precursors and VOCs. In this study, the growth patterns of *G. potus* varied depending on the co-cultured species. *G.potus* generally grew more slowly in groups lacking L. *plantarum*, suggesting an affinity for lactic acid or acidic environments. *G. potus* strains have been reported to grow at pH 3.6, indicating their general acid tolerance (Sombolestani et al., 2019). An exception was observed in groups containing only *H. uvarum*, *G. potus* demonstrated efficient metabolism and substantial acetic acid production. This phenomenon may be attributed to lower sugar consumption rate of *H. uvarum*, which could provide a more sustained supply of metabolites for *G. potus*, coupled with the latter's known ethanol tolerance (Sombolestani et al., 2019). Similar observations by Diaz-Munoz et al. (2023) revealed that the limited sugar consumption capacity of *H. opuntiae*, combined with the absence of pulp liquefaction, led to excessive AAB growth and fermentation failure. These observations underscore the importance of balanced microbial interactions in successful cocoa fermentation. During spontaneous box fermentation of cocoa beans, AAB proliferate significantly due to the oxygen introduced through gaps formed by the liquefaction of cocoa pulp (Farrera et al., 2021). However, in the pure pulp medium, *G. potus*, introduced from the start, can still grow and metabolize effectively.

While some researchers argue that LAB are not essential for successful cocoa fermentation or the development of unique cocoa flavors (Ho et al., 2015), we found that LAB growth is positively associated with ester compounds such as methyl hexanoate and octyl acetate (Fig. S1). LAB has also been shown to demonstrate the potential to convert alcohols and acids into esters (López-Salas et al., 2022), possibly through the catalytic action of carboxylesterases (Paradhipta et al., 2021). Beyond their independent flavor development capabilities, the synergistic interaction between LAB and yeasts plays a crucial role. For instance, co-inoculation of *Lactobacillus fermentum* 223 and *S. cerevisiae* H290 directly improved the sensory attributes of cocoa beans (Romanens et al., 2020). Notably, the presence of small amounts of acetic acid in groups initially devoid of AAB may be attributed to LAB's capability to convert citric acid into acetic acid under acidic conditions (Lefeber et al., 2011). However, further investigation is needed regarding the antifungal properties of LAB in our study, which has been identified as a key characteristic in many cocoa-related LAB studies (Das Neves Selis et al., 2021; Korcari et al., 2022).

Acetic acid and acetate, the primary products of AAB, significantly influence the development of desirable cocoa flavors. The newly discovered species *G. potus*, which is present in fruits and fermented food products, has the ability to produce aldehydes and ketones from sugars and alcohols (Wang, Rutherfurd-Markwick, et al., 2022). As the only AAB in this investigation, it was substantially linked to ethyl esters and acetates (*p* < 0.05) (Fig. S1). Notably, *G. potus* produced substantial amounts of acetic acid when only yeast *H. uvarum* was present (especially with LAB), likely due to the slower sugar consumption rate of *Hanseniaspora*. The excessive acidity might result in undesirable off-flavors.

As mentioned above, the fermentation process involves various microorganisms that can alter the chemical composition of VOCs. These interactions might lead to the new compound formation or new modifications of already existing ones, possessing unexpected aroma profiles. The scalability and flexibility of machine learning algorithms allow for the analysis of complex datasets to reveal patterns and relationships that may not be easily discernible through traditional methods (Bzdok et al., 2018). The simple unsupervised machine learning method PCA did not adequately explain the distribution of flavor compounds across multiple groups of fermented pulp (PC1 + PC2 = 42.69 %), suggesting the need to consider some complex modeling approaches. In small-scale, experiment-based data on cocoa pulp fermentation, we used 19 methods to preliminarily probe into the mixtures of VOCs and their association with a set of olfactory characteristics. During cross-validation, the model quality exceeded 0.7 (except linear regression) without the risk of overfitting (no fold was over 0.9), and RF and GBM had the best performance. These results show that decision tree-based algorithms could deal better with high-dimensional data, complex interaction, noise reduction, and hence make good predictions(Breiman, 2001; Friedman, 2002). Quantifying feature importance also helps explain the potential logical relationships between inputs and targets (Colantonio et al., 2022).

**Fig. 5:**
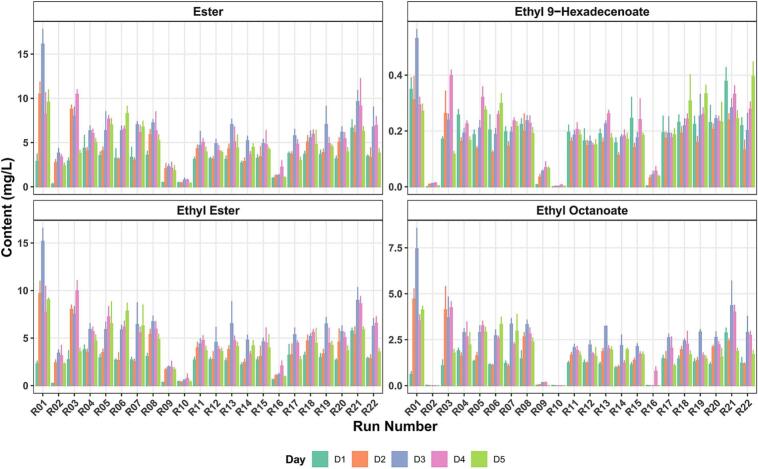
Changes in selected VOCs (total esters, ethyl esters, ethyl 9-hexadecenoate, and ethyl octanoate) during fermentation period (D1-D5) across 22 experimental runs.

VOCs formed in cocoa pulp can diffuse into cocoa beans and participate in the development of bean flavor. The inoculation of starters can enhance this diffusion further. (Besançon et al., 2024). Yeasts can produce abundant ethyl esters and acetates, improving the sensory quality of fermentation, which corresponds to results linked to *H. thailandica* and *Pichia kluyveri* (Mendoza Salazar et al., 2022). Additionally, cocoa pulp may contain alcohol acyltransferases or carboxylesterases involved in ester formation (Martínez-Rivas et al., 2022). Ethyl octanoate and ethyl 9-hexadecenoate are two ethyl esters that contribute to overall preference in cocoa pulp, playing significant roles in the aroma profiles of other products. The concentrations of both ethyl esters and total esters were lower in fermentations where *Hanseniaspora* was the only yeast present (Fig. 5). Ethyl octanoate has been described as an extremely important ester in the aroma profile of spirits (Wang, Guo, et al., 2022). Ethyl 9-hexadecenoate is significant in the VOC fingerprint of wine, responsible for reducing the acidity of the product (Zhong et al., 2021), and it can be supposed as a unique VOC of indigenous Hainan cocoa yeasts. Ethyl palmitate and ethyl acetate contribute to the savory characteristics of cocoa pulp. These compounds do not solely build aroma; they can change the release behaviors of VOCs, increase their olfactory detection thresholds (ODTs), and influence the overall aroma profile (Guo et al., 2024). Lactic acid bacteria may help produce phenylacetic acid ethyl ester, giving cocoa a mild sweetness (Xu et al., 2023). According to Bickel Haase et al. (2021), unsaturated fatty acids can be present in a small amount in cocoa pulp. Medium-chain fatty acids, such as capric acid, can contribute to a pleasant aroma, despite often being characterized as pungent in flavor descriptions. As well as the microbial contribution, VOCs and flavor precursors are sensitive to heat treatment, where, for example, 5-methyl furfural may originate from UHT (Bickel Haase et al., 2023). Overall, the aroma profile of food products is not solely determined by individual compounds but rather by the complex interactions among various components, suggesting the effectiveness of machine learning algorisms for the discovery of the connections between VOCs and olfactory perceptions.

Starter cultures have been instrumental in improving pulp drainage, accelerating cocoa pulp-bean mass fermentation, and enhancing the overall quality of cured and roasted cocoa beans, ultimately impacting the quality of chocolates derived from these beans (Díaz-Muñoz et al., 2021). The four microbial strains we employed were all sourced from the natural fermentation of Hainan-flavored cocoa (Chang et al., 2023). By targeting the overall preference score and selected metabolites, we used the fermentation agent ratio as input for polynomial or neural network modeling. After optimization using GA, we obtained the optimal solution, which was then evaluated using GBDT for better model quality. To prevent excessive constraints from pushing the optimization results toward the boundary, we did not impose the restriction that yeast must account for more than 50 % during GA optimization. Nevertheless, the results still emphasized the positive impact of yeasts. GBDT performed excellently in handling noisy data and complex problems. Its robustness allows it to effectively manage outliers or noise in the data, reducing the risk of significant prediction errors due to local disturbances (Li et al., 2020). The validation results of the fermentation agent composition using GBDT also enhanced the reliability of polynomial regression and Neural Network modeling.

Although this study used machine learning methods to identify VOCs affecting the olfactory characteristics of cocoa pulp, several limitations should be noted. The sample independence may be compromised as multiple samples were collected from the same fermentation mixture at different time points. The well-performed decision tree models RF and GBM in this study may also subsequently be able to analyze the direction of relationships through SHapley Additive exPlanations (SHAP) (Lundberg, 2017). Neural network modeling with limited sample size (*n* = 22) poses a potential risk of overfitting in starter optimization, and GBDT validation based on GA optimization should also be considered for its limitations in predicting extreme values. Further validation through larger scale box fermentation experiments is ongoing to verify these findings and elucidate the cross-linking mechanisms between pulp and beans. Sequencing targeting yeast, rather than culture-dependent methods, can more clearly elucidate interactions between yeasts.

Additionally, since this study focused solely on Trinitario fine flavor cocoa from Hainan, future research should investigate different cocoa varieties and geographical origins to establish more generalizable findings and understand how genetic and environmental factors influence VOC development during fermentation.

## 5 Conclusion

In this study, the relationship between aroma compounds and olfactory attributes in cocoa pulp fermentation were evaluated by different machine learning techniques. We then investigated the differences in fermentation performance among various combinations of yeasts, LAB, and AAB. All algorithms, except for linear regression, demonstrated considerable average explanatory ability, with GBM and RF showing particularly outstanding predictive performance for acid characteristics (R^2^ = 0.85). Through GBM and Bayes models, ethyl esters, including ethyl octanoate and ethyl 9-decenoate, and alcohols were found to have a constructive impact on the sensory panel, while acetic acid significantly impairs flavor. The importance of yeast, and the unique flavor potential of LAB and AAB were reaffirmed through polynomial regression and MLP, optimized by GA and validated by GBDT, highlighting the application of mixed-culture starters. However, further research is needed to explore the interacting mechanisms among microorganisms and the impact of these findings on farm-scale cocoa fermentation.

## CRediT authorship contribution statement

**Haode Chang:** Writing – original draft, Software, Methodology, Conceptualization. **Chunhe Gu:** Methodology, Data curation, Conceptualization. **Quanmiao Zhang:** Validation, Formal analysis. **Wenjing Zhang:** Investigation. **Liru Ma:** Visualization. **Fei Liu:** Supervision. **Zhen Feng:** Writing – review & editing, Resources, Funding acquisition.

## Declaration of competing interest

The authors declare that they have no known competing financial interests or personal relationships that could have appeared to influence the work reported in this paper.

## Data Availability

Data will be made available on request.

## Appendix A Supplementary data

Supplementary material 1 Image 1

Supplementary material 2 Image 2

Supplementary material 3 Image 3

Supplementary material 4 Image 4
