# Supplementary material for: Investigation of aromatic compounds and olfactory profiles in cocoa pulp fermentation using yeast-based starters: A Volatilomics and machine learning approach
Source: Food Chem X. 2025 Feb 25;26:102315. doi: 10.1016/j.fochx.2025.102315 (PMC11914200; doi:10.1016/j.fochx.2025.102315)
Supplement: Supplementary file 1 — Supplementary material 1 [file mmc1.docx]

**Table S1** Performance metrics of 19 machine learning models using 10-fold cross-validation: Accuracy and mean squared error (MSE) for predicting sensory attributes

| Model | Accuracy | | | | | | MSE | | | | | |
| --- | --- | --- | --- | --- | --- | --- | --- | --- | --- | --- | --- | --- |
|  | Average | Overall preference | Sweet | Sour | Herbaceous | Savory | Average | Overall preference | Sweet | Sour | Herbaceous | Savory |
| BayesA | 0.73 | 0.6823 | 0.7166 | 0.7897 | 0.7097 | 0.7516 | 0.5043 | 0.5639 | 0.5247 | 0.3707 | 0.5513 | 0.511 |
| BayesB | 0.7326 | 0.6816 | 0.7101 | 0.7971 | 0.713 | 0.7612 | 0.5036 | 0.5657 | 0.5325 | 0.3865 | 0.5464 | 0.4868 |
| BayesC | 0.7322 | 0.6788 | 0.7102 | 0.7894 | 0.7191 | 0.7636 | 0.5027 | 0.5693 | 0.5318 | 0.3947 | 0.5336 | 0.4839 |
| Bayesian Ridge | 0.7185 | 0.6816 | 0.7158 | 0.7382 | 0.707 | 0.75 | 0.5237 | 0.5712 | 0.5289 | 0.4481 | 0.5538 | 0.5168 |
| Bayesian Lasso | 0.7321 | 0.687 | 0.7204 | 0.7857 | 0.7073 | 0.7601 | 0.4969 | 0.5503 | 0.5179 | 0.3747 | 0.5507 | 0.4911 |
| Random Forest | 0.7716 | 0.7619 | 0.7531 | 0.8543 | 0.723 | 0.7657 | 0.4178 | 0.4122 | 0.4424 | 0.2719 | 0.5217 | 0.4407 |
| SVM Linear | 0.6844 | 0.6308 | 0.6635 | 0.7038 | 0.6499 | 0.7739 | 0.6673 | 0.717 | 0.7168 | 0.6778 | 0.7473 | 0.4777 |
| Elastic Net | 0.7118 | 0.6604 | 0.6671 | 0.7545 | 0.7188 | 0.7581 | 0.5854 | 0.6107 | 0.7801 | 0.5117 | 0.543 | 0.4817 |
| Neural Net | 0.7417 | 0.7117 | 0.7439 | 0.7403 | 0.7234 | 0.7891 | 0.4716 | 0.5149 | 0.4787 | 0.4358 | 0.5153 | 0.4133 |
| Kernel PLS | 0.7034 | 0.7067 | 0.6992 | 0.6798 | 0.6826 | 0.7485 | 0.543 | 0.5073 | 0.5476 | 0.5537 | 0.5889 | 0.5176 |
| Linear Regression | 0.3949 | 0.3726 | 0.2773 | 0.3444 | 0.4637 | 0.5167 | 11.9466 | 10.6371 | 15.2718 | 13.3518 | 10.3877 | 10.0842 |
| RKHS | 0.72 | 0.684 | 0.7189 | 0.7379 | 0.707 | 0.7521 | 0.5201 | 0.563 | 0.5215 | 0.4499 | 0.5533 | 0.5126 |
| Gradient Boost Machine | 0.775 | 0.7684 | 0.7381 | 0.8516 | 0.747 | 0.7698 | 0.4092 | 0.4015 | 0.4643 | 0.2671 | 0.4761 | 0.437 |
| Relevent Vector Machine | 0.7239 | 0.7011 | 0.6956 | 0.6884 | 0.7786 | 0.7556 | 0.4951 | 0.5149 | 0.5571 | 0.5423 | 0.4265 | 0.4349 |
| SVM Radial | 0.7242 | 0.716 | 0.7278 | 0.6283 | 0.765 | 0.7841 | 0.5044 | 0.5008 | 0.4924 | 0.6587 | 0.4634 | 0.4065 |
| SVM Radial Sigma | 0.7336 | 0.7068 | 0.7573 | 0.6524 | 0.7531 | 0.7986 | 0.4832 | 0.4948 | 0.4436 | 0.6131 | 0.4771 | 0.3876 |
| Bayesian Neural Net | 0.6732 | 0.7092 | 0.7006 | 0.646 | 0.6806 | 0.6295 | 0.7094 | 0.535 | 0.5589 | 0.7936 | 0.7696 | 0.8898 |
| LASSO | 0.7122 | 0.6646 | 0.6507 | 0.762 | 0.7156 | 0.7681 | 0.6045 | 0.5968 | 0.9109 | 0.5033 | 0.5448 | 0.4668 |
| XGBoost | 0.7377 | 0.719 | 0.6832 | 0.8371 | 0.7154 | 0.7338 | NA | NA | NA | NA | NA | NA |

*NA: not available
