# Supplementary material for: Investigation of aromatic compounds and olfactory profiles in cocoa pulp fermentation using yeast-based starters: A Volatilomics and machine learning approach
Source: Food Chem X. 2025 Feb 25;26:102315. doi: 10.1016/j.fochx.2025.102315 (PMC11914200; doi:10.1016/j.fochx.2025.102315)
Supplement: Supplementary file 2 — Supplementary material 2 [file mmc2.docx]

**
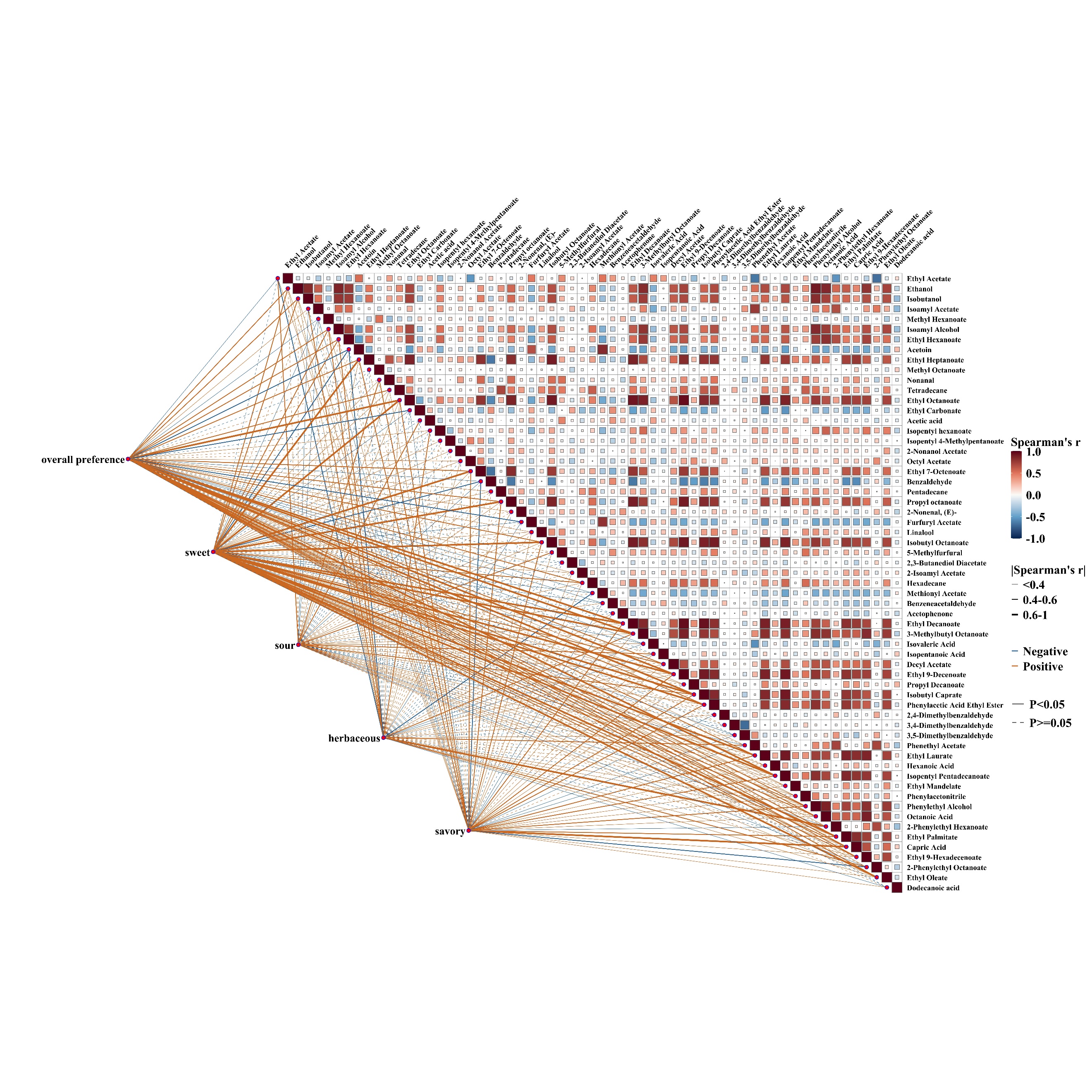
Figure S1**

Spearman correlation analysis of VOCs and olfactory attributes in cocoa pulp fermentation without filtering.
