# Supplementary material for: Investigation of aromatic compounds and olfactory profiles in cocoa pulp fermentation using yeast-based starters: A Volatilomics and machine learning approach
Source: Food Chem X. 2025 Feb 25;26:102315. doi: 10.1016/j.fochx.2025.102315 (PMC11914200; doi:10.1016/j.fochx.2025.102315)
Supplement: Supplementary file 3 — Supplementary material 3 [file mmc3.docx]

Sample No.______ Date. ________

Name ________ Today’s No. ________

**Cocoa Pulp Odor Evaluation Form**

**Sweet**

Weak |-----------------------|------------------------|------------------------|------------------------| Strong

**Caramel**

Weak |-----------------------|------------------------|------------------------|------------------------| Strong

**Chocolate**

Weak |-----------------------|------------------------|------------------------|------------------------| Strong

**Honey**

Weak |-----------------------|------------------------|------------------------|------------------------| Strong

**Fruity**

Weak |-----------------------|------------------------|------------------------|------------------------| Strong

**Pungent**

Weak |-----------------------|------------------------|------------------------|------------------------| Strong

**Sour**

Weak |-----------------------|------------------------|------------------------|------------------------| Strong

**Lactic**

Weak |-----------------------|------------------------|------------------------|------------------------| Strong

**Citrusy**

Weak |-----------------------|------------------------|------------------------|------------------------| Strong

**Fresh**

Weak |-----------------------|------------------------|------------------------|------------------------| Strong

**Green**

Weak |-----------------------|------------------------|------------------------|------------------------| Strong

**Plant**

Weak |-----------------------|------------------------|------------------------|------------------------| Strong

**Cheesy**

Weak |-----------------------|------------------------|------------------------|------------------------| Strong

**Fatty**

Weak |-----------------------|------------------------|------------------------|------------------------| Strong

**Umami**

Weak |-----------------------|------------------------|------------------------|------------------------| Strong

**Overall Impression**

Bad |-----------------------|------------------------|------------------------|------------------------| Good

(The following content is not included in this form.)

Panelists are required to complete 5 tests simultaneously and draw a short line ‘|’ on the line using a 0.5 mm ballpoint pen.

The original language of the form is Chinese. In descriptive language for flavors, different languages may have subtle differences in defining the same aroma (Majid et al., 2018).

Majid, A., Roberts, S. G., Cilissen, L., Emmorey, K., Nicodemus, B., O’Grady, L., Woll, B., LeLan, B., de Sousa, H., Cansler, B. L., Shayan, S., de Vos, C., Senft, G., Enfield, N. J., Razak, R. A., Fedden, S., Tufvesson, S., Dingemanse, M., Ozturk, O., Brown, P., Hill, C., Le Guen, O., Hirtzel, V., van Gijn, R., Sicoli, M. A., & Levinson, S. C. (2018). Differential coding of perception in the world’s languages. *Proceedings of the National Academy of Sciences*, *115*(45), 11369-11376. <https://doi.org/10.1073/pnas.1720419115>
